# Immune Checkpoint Protein Expression Defines the Prognosis of Advanced Thyroid Carcinoma

**DOI:** 10.3389/fendo.2022.859013

**Published:** 2022-04-27

**Authors:** Yi Luo, Yi-Chen Yang, Cen-Kai Shen, Ben Ma, Wei-Bo Xu, Qi-Feng Wang, Yan Zhang, Tian Liao, Wen-Jun Wei, Yu Wang

**Affiliations:** ^1^Department of Oncology, Shanghai Medical College, Fudan University, Shanghai, China; ^2^Department of Head and Neck Surgery, Shanghai Cancer Center, Fudan University, Shanghai, China; ^3^Department of Pathology, Shanghai Cancer Center, Fudan University, Shanghai, China

**Keywords:** immunotherapy, immune checkpoint, anaplastic thyroid carcinoma, poorly differentiated thyroid carcinoma, locally advanced papillary thyroid carcinoma

## Abstract

**Background:**

Patients with advanced thyroid carcinoma (TC), such as anaplastic thyroid carcinoma (ATC), poorly differentiated thyroid carcinoma (PDTC), and locally advanced papillary thyroid carcinoma (PTC), have poor prognoses and require novel treatments. Immune checkpoint (ICP) inhibitors have demonstrated encouraging and good results; nevertheless, their effect in advanced TCs remains largely unclear. Thus, we demonstrated ICP profiles and investigated their potential clinical significance.

**Methods:**

A total of 234 TC patients were involved, with 22 ATCs, 44 PDTCs, and 168 PTCs, including 58 advanced PTCs. Immunohistochemistry was performed to evaluate nine ICPs [programmed cell death ligand 1 (PDL1), Programmed cell death 1 (PD1), cytotoxic T lymphocyte-associated protein 4 (CTLA4), B and T lymphocyte attenuator (BTLA), T-cell immunoglobulin and immunoreceptor tyrosine-based inhibitory motif (ITIM) domain (TIGIT), lymphocyte activation gene 3 (LAG3), V-domain immunoglobulin suppressor of T-cell activation (VISTA), B7 homolog 3 (B7-H3), and T-cell immunoglobulin and mucin domain- 3 protein (TIM3)] expression *via* tissue microarrays (TMAs), and clinical correlations were analyzed simultaneously.

**Results:**

ATC had the highest positive rate of ICPs among the three pathological types, as well as relatively high ICP co-expression. ATC with high expression of PDL1 positivity had a poor prognosis. Shorter survival was associated with VISTA, B7H3, TIM3, and TIGIT expression in PDTC. The greater the co-expression of these four ICPs, the poorer the prognosis in PDTC patients. VISTA and B7H3 were the two most commonly expressed ICPs in advanced PTC, both of which were linked to a poor prognosis.

**Conclusions:**

PDL1 is linked to the overall survival (OS) of ATC. A subset of PDTC is likely immunogenic with poor prognosis and co-expression of VISTA, B7H3, TIM3, and TIGIT. Furthermore, VISTA and B7H3 are prognostic biomarkers in advanced PTC. Single or combined blockade targeting these ICPs might be effective for advanced TCs in the future.

## Introduction

Thyroid carcinoma (TC) has increased dramatically during the last several decades. The majority of TCs are non-medullary thyroid carcinoma (NMTC) with well differentiation such as papillary thyroid carcinoma (PTC) and follicular thyroid carcinoma (FTC) and have a good prognosis. However, there are some patients diagnosed as having advanced TC based on the differential grade and local extension involvement. Firstly, anaplastic thyroid carcinoma (ATC) is one of the most lethal malignancies, with a median survival of 6–10 months from the time of diagnosis (1–3). Secondly, poorly differentiated thyroid carcinoma (PDTC) is a subgroup of differentiated thyroid carcinoma (DTC) with morphological and biological characteristics intermediate between the well-differentiated TC (WDTC) and ATC, as determined by the assessment of selected histomorphologic features included in the Turin criteria), and its presence significantly reduces 5-year survival to less than 20% (4, 5). Thirdly, according to the 8th American Joint Committee on Cancer (AJCC), locally advanced WDTC is a notable subtype with a significant challenge of extensive resection of local lesions, particularly when local invasion of the trachea or esophagus exists. Therapeutic options for patients displaying unresectable WDTC tumors remain limited. Unfortunately, standard therapies for these advanced TCs are limited. As a result, it is critical to investigate reasonable and individualized therapy solutions.

Over the past few decades, immunotherapeutics have shown great efficacy (6, 7). Immune checkpoint inhibitors (ICIs) have been developed to rejuvenate the antitumor response of immune cells. At present, programmed cell death 1/programmed cell death ligand 1 (PD1/PDL1) and cytotoxic T lymphocyte-associated protein 4 (CTLA4) are the most well-studied signals and have shown impressive therapeutic benefits in different cancers (8–10). For TC patients with advanced disease, it has been shown that ICIs have potential in the future but are not currently effective enough (11–14). Moreover, there are many other novel immune checkpoints (ICPs) being studied, such as T-cell immunoglobulin and immunoreceptor tyrosine-based inhibitory motif (ITIM) domain (TIGIT), lymphocyte activation gene 3 (LAG3), and V-domain immunoglobulin suppressor of T-cell activation (VISTA). For instance, small molecules against VISTA have been demonstrated to have acceptable tolerability profiles and clinical activity (15). Whereas in terms of the precise and comprehensive treatment of advanced TC, the therapeutic value of ICIs needs to be investigated further. Combination of ICIs might be promising, as one recent study found that the co-inhibition of TIGIT and PDL1 could improve the clinical outcome of tumor patients (16). To summarize, greater evidence of expressive ICP profiles and synergy between various ICPs is urgently needed to enhance single-drug regimens. In this work, we showed the immunological landscape of a group of ICPs, including PDL1, PD1, TIGIT, LAG3, VISTA, B7H3, CTLA4, and T-cell immunoglobulin domain and mucin domain 3 (TIM3) and attempted to investigate the predictive biomarkers of aggressive TCs.

## Materials and Methods

### Patient Demographics and Clinicopathologic Variables

A total of 234 patients were enrolled including 168 PTCs, 44 PDTCs, and 22 ATCs. Regarding the PTC, only classical subtype and follicular variant were enrolled from August 2006 to July 2020 at Fudan University Shanghai Cancer Center (FUSCC). Based on the 8th AJCC, PTC patients with obvious invasion of recurrent laryngeal nerve, larynx, trachea, esophagus, common carotid artery, or internal jugular vein were confirmed as locally advanced disease (pT4 stage), and further analysis was conducted in this subset. PDTC was diagnosed based on the Turin proposal (5). Patients with PTC, PDTC, and ATC underwent standard treatments including thyroidectomy and routine lymph node dissection (central and/or lateral lymph node dissection), followed by adjuvant therapies, if indicated, such as radioiodine, radiotherapy, chemotherapy, and radiotherapy with concurrent chemotherapy. No patient received immunotherapies. All samples, as certified by two pathologists (Q-FW, YZ), were collected during the surgeries, which could avoid any interferences from the postoperative adjuvant therapies. Clinicopathological information was evaluated by reviewing the medical records, including age, gender, histologic type, tumor size, tumor-node-metastasis (TNM) stage, disease relapse, and survival data. Patients who were lost to follow-up were excluded from the survival analysis. And patients with follow-up less than 1 month were also excluded in order to reduce statistical bias. The study was approved by the institutional review board of FUSCC (050432-4-1911D), and informed consent was provided for each patient.

### Tissue Microarrays and Immunohistochemistry of Immune Checkpoints

A tissue microarray (TMA) of tumor tissue was constructed. Briefly, formalin-fixed, paraffin-embedded (FFPE) tissue blocks from resected TC were obtained. Tissue cylinders (1.5 mm) were punched from representative tissue areas of each tissue block and brought into one recipient paraffin block, which was then cut serially (4 μm). To overcome the heterogeneity expression, we performed TMA using 4–7 representative cores that included at least 50% tumor cells. Deparaffinization of histological sample slides was performed with xylene and different concentrations of ethanol and washed with phosphate-buffered saline (PBS). Next, the slides were incubated with 3% hydrogen peroxidase at room temperature, and 5% goat serum was applied to block the nonspecific binding. Immunohistochemical staining was performed at 4°C overnight using primary antibodies (**Supplementary Table S1**). Then, the slides were washed with PBS and probed with secondary antibody for 2 h at 37°C. Subsequently, the slides were stained with Diaminobenzidine (DAB) and hematoxylin, followed by dehydration. KF-PRO-120 Digital Scanner and K-Viewer System (Konfoong Biotech, Ningbo, China) were used to view the screen slides.

### Evaluation of Immune Checkpoint Immunohistochemistry Staining

The expression of ICPs was semiquantitatively assessed as previously published (17–28). Briefly, the expression of PDL1, PD1, CTLA4, BTLA, TIGIT, LAG3, VISTA, B7H3, and TIM3 on tumor-infiltrating immune cells (TIICs) and tumor cells was evaluated based on the combined positive score (CPS), defined as the percentage of positive tumor cells (total/partial membrane staining) and tumor TIICs (membrane/cytoplasm staining) relative to the total number of tumor cells (18, 29). The median CPS of cores of each sample was used as the final CPS. ICP expression was further stratified into negative (CPS <1), weak (1 ≤ CPS < 10), moderate (10 ≤ CPS < 30), and strong (CPS ≥30). The results of immunostained slides were determined by two experienced pathologists (Q-FW, YZ) blinded to the clinical outcome, and discrepant results were resolved by consensus review.

### Statistical Analysis

Statistical significance of continuous parameters was determined using Student’s t test and Kruskal–Wallis test. Meanwhile, the chi-square test or Fisher’s exact test was used to analyze the statistical significance of categorical parameters. The association between the expression of ICPs was evaluated by the Spearman correlation. The overall survival (OS) and disease-free survival (DFS) curves were drawn by the Kaplan–Meier (KM) method. Univariate and multivariate Cox regression were used to investigate prognostic characteristics. All statistics were two‐tailed, and *p* < 0.05 was considered statistically significant. All analyses were performed using R (version 4.1.0).

## Results

### Patient Characteristics

In the study, 234 patients with thyroid cancer were enrolled, including 22 ATCs, 44 PDTCs, and 168 PTCs. The basic clinical variables were summarized in **Table 1**. In the overall cohort, 122 were women and 112 were men. The median age at the time of diagnosis was 56.00, 55.50, and 63.50 years in PTC, PDTC, and ATC groups, respectively. In addition, 34.5% (58/168) PTC patients were classified as locally advanced disease (pT4). Lymph node metastasis (LNM) was common in our study, which was observed in more than half of patients of each subtype. In this study, 18.2% (4/22) ATCs developed distant metastasis, while it was significantly low in PTC and PDTC, with 6.0% and 9.1%, respectively. Among these 234 patients, 53 died during follow-up, which included 17 ATCs, 26 PDTCs, and 10 PTCs.

**Table 1:** Baseline characteristic of 234 patients with thyroid carcinoma.

		PTC	PDTC	ATC
**Number**		168	44	22
**Gender (%)**	Women	86 (51.2)	28 (63.6)	8 (36.4)
	Men	82 (48.8)	16 (36.4)	14 (63.6)
**Age, (years)**		56.00 (42.00, 63.00)	55.50 (41.25, 66.50)	63.50 (53.75, 66.50)
**Tumor size (cm), median (range)**		2.70 (1.58, 3.50)	2.60 (1.55, 4.50)	3.50 (2.55, 6.75)
**T Stage (%)**	T1	43 (25.6)	13 (29.5)	0 (0.0)
	T2	33 (19.6)	2 (4.5)	5 (22.7)
	T3	34 (20.2)	16 (36.4)	5 (22.7)
	T4	58 (34.5)	13 (29.5)	12 (54.5)
**N Stage (%)**	N0	35 (20.8)	9 (20.5)	6 (27.3)
	N1a	38 (22.6)	7 (15.9)	2 (9.1)
	N1b	94 (56.0)	28 (63.6)	11 (50.0)
	Nx	1 (0.6)	0 (0.0)	3 (13.6)
**M Stage (%)**	M0	158 (94.0)	40 (90.9)	18 (81.8)
	M1	10 (6.0)	4 (9.1)	4 (18.2)
**Death (%)**	Alive	150 (89.3)	16 (36.4)	4 (18.2)
	Dead	10 (6.0)	26 (59.1)	17 (77.3)
	NA^*^	8 (4.7)	2 (4.5)	1 (4.5)

*Not applicable.

### Expression Landscape of Immune Checkpoints

**Figure 1:**
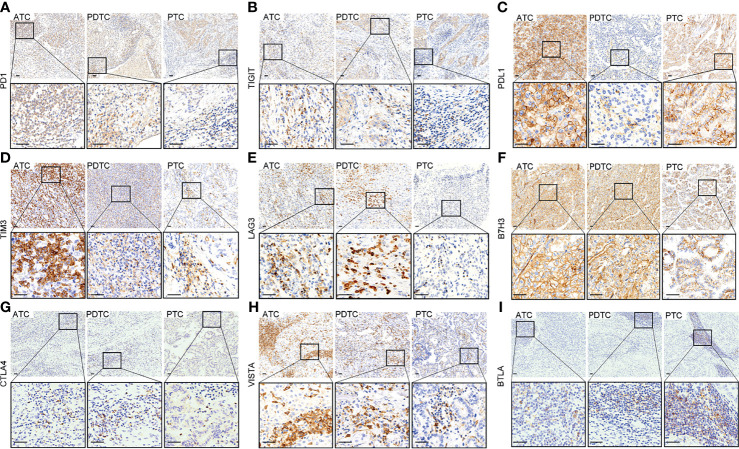
Representative immunohistochemical staining of positive expression of PD1 **(A)**, TIGIT **(B)**, PDL1 **(C)**, TIM3 **(D)**, LAG3 **(E)**, B7H3 **(F)**, CTLA4 **(G)**, VISTA **(H)**, and BTLA **(I)** in anaplastic thyroid carcinoma (ATC), poorly differentiated thyroid carcinoma (PDTC), and papillary thyroid carcinoma (PTC), respectively (upper: ×100, lower: ×400, scale bar: 40 μm).

**Figure 2:**
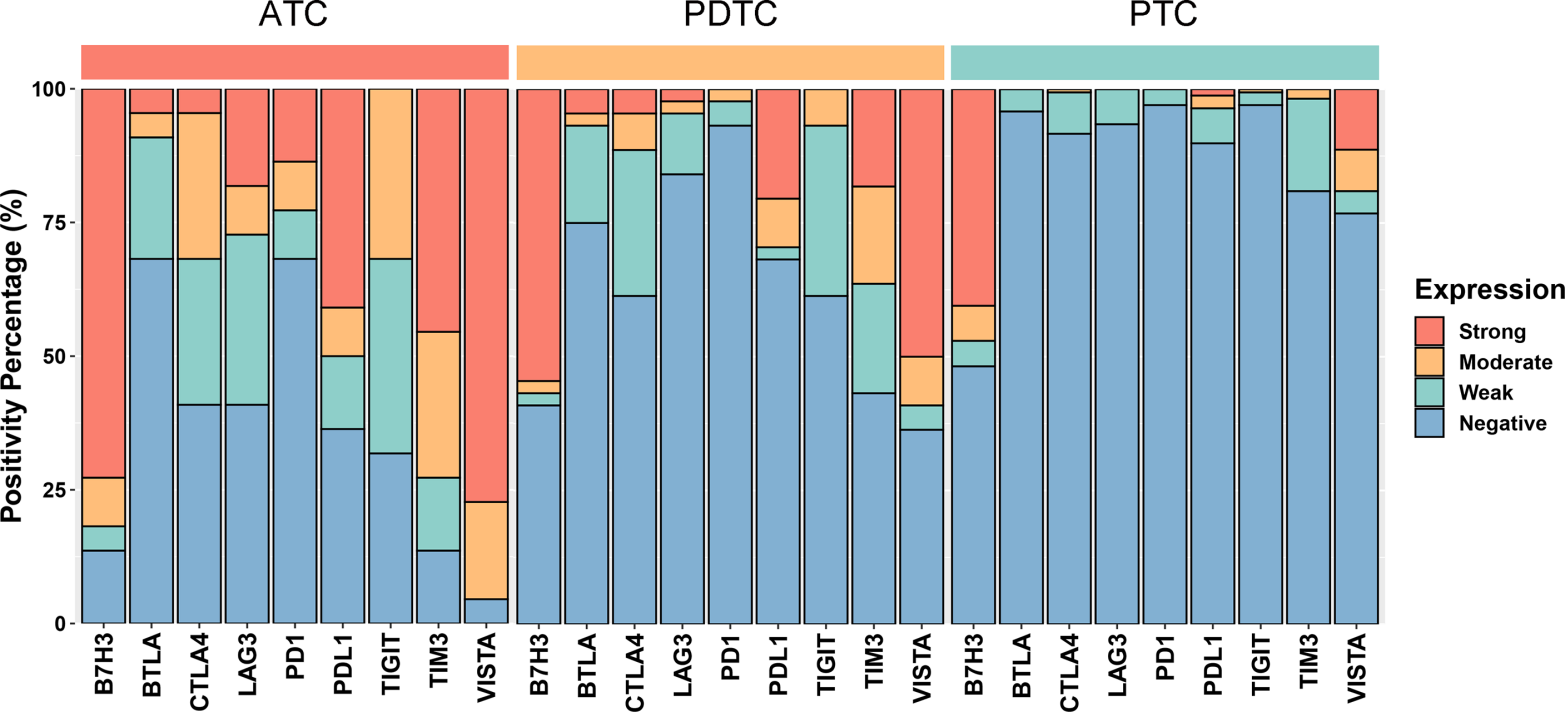
Bar plots showing the expression profiles of PDL1, PD1, CTLA4, BTLA, TIGIT, LAG3, VISTA, B7H3, and TIM3 in anaplastic thyroid carcinoma (ATC), poorly differentiated thyroid carcinoma (PDTC), and papillary thyroid carcinoma (PTC), respectively. According to the chi-square test or Fisher’s exact test if indicated, the p values of the expression proportion of all checkpoints are less than 0.001, except B7H3, which is equal to 0.025.

The representative regions of IHC are shown in **Figure 1**. The distributions of ICPs were significantly different among cancer histology types (**Figure 2** and **Table 2**). PTC showed relatively negative to low expression of almost all nine ICPs, whereas ATC exhibited frequently moderate to strong expression of ICPs, and the expressed ICP profiles of PDTC were intermediate between ATC and PTC (**Figure 2** and **Table 2**). For instance, 63.6% ATC, 31.8% PDTC, and 10.1% PTC had positive expression of PDL1, respectively (**Figure 2** and **Table 2**). In PTC cases, the positivity of PDL1 expression was generally weak (**Figure 2** and **Table 2**). However, most PDL1-positive ATCs and PDTCs exhibited strong expression (**Figure 2** and **Table 2**). The ratio of PD1 positivity was much lower among the three pathological subgroups compared with PDL1, of which 97.0% PTCs, 93.2% PDTCs, and 68.2% ATCs showed PD1 negativity. Notably, B7H3 was the highest positive ICP expression, with 40.5%, 54.5%, and 72.7% strong expression in PTC, PDTC, and ATC cohorts, respectively, followed by VISTA that exhibited substantially strong positivity in 77.3% ATC and 50.0% PDTC (**Figure 2** and **Table 2**). Besides, no expression of BTLA, CTLA4, LAG3, TIGIT, and TIM3 was observed in 68.2%, 40.9%, 40.9%, 31.8%, and 13.6% of ATC, 75.0%, 61.4%, 84.1%, 61.4%, and 43.2% of PDTC, and 95.8%, 91.7%, 93.5%, 97.0%, and 81.0% of PTC, respectively (**Figure 2** and **Table 2**).

**Table 2:** The expression proportion of immune checkpoints in thyroid carcinoma.

Immune checkpoint	Level	Overall	ATC	PDTC	PTC	*p* value	Test
		n = 234	n = 22	n = 44	n = 168		
**PDL1 (%)**	Negative	189 (80.8)	8 (36.4)	30 (68.2)	151 (89.9)	<0.001	Fisher’s exact test
	Weak	15 (6.4)	3 (13.6)	1 (2.3)	11 (6.5)		
	Moderate	10 (4.3)	2 (9.1)	4 (9.1)	4 (2.4)		
	Strong	20 (8.5)	9 (40.9)	9 (20.5)	2 (1.2)		
**PD1 (%)**	Negative	219 (93.6)	15 (68.2)	41 (93.2)	163 (97.0)	<0.001	Fisher’s exact test
	Weak	9 (3.8)	2 (9.1)	2 (4.5)	5 (3.0)		
	Moderate	3 (1.3)	2 (9.1)	1 (2.3)	0 (0.0)		
	Strong	3 (1.3)	3 (13.6)	0 (0.0)	0 (0.0)		
**TIGIT (%)**	Negative	197 (84.2)	7 (31.8)	27 (61.4)	163 (97.0)	<0.001	Chi-square test
	Weak	26 (11.1)	8 (36.4)	14 (31.8)	4 (2.4)		
	Moderate	11 (4.7)	7 (31.8)	3 (6.8)	1 (0.6)		
**LAG3 (%)**	Negative	203 (86.8)	9 (40.9)	37 (84.1)	157 (93.5)	<0.001	Fisher’s exact test
	Weak	23 (9.8)	7 (31.8)	5 (11.4)	11 (6.5)		
	Moderate	3 (1.3)	2 (9.1)	1 (2.3)	0 (0.0)		
	Strong	5 (2.1)	4 (18.2)	1 (2.3)	0 (0.0)		
**VISTA (%)**	Negative	146 (62.4)	1 (4.5)	16 (36.4)	129 (76.8)	<0.001	Fisher’s exact test
	Weak	9 (3.8)	0 (0.0)	2 (4.5)	7 (4.2)		
	Moderate	21 (9.0)	4 (18.2)	4 (9.1)	13 (7.7)		
	Strong	58 (24.8)	17 (77.3)	22 (50.0)	19 (11.3)		
**B7H3 (%)**	Negative	102 (43.6)	3 (13.6)	18 (40.9)	81 (48.2)	0.025	Fisher’s exact test
	Weak	10 (4.3)	1 (4.5)	1 (2.3)	8 (4.8)		
	Moderate	14 (6.0)	2 (9.1)	1 (2.3)	11 (6.5)		
	Strong	108 (46.2)	16 (72.7)	24 (54.5)	68 (40.5)		
**CTLA4 (%)**	Negative	190 (81.2)	9 (40.9)	27 (61.4)	154 (91.7)	<0.001	Fisher’s exact test
	Weak	31 (13.2)	6 (27.3)	12 (27.3)	13 (7.7)		
	Moderate	10 (4.3)	6 (27.3)	3 (6.8)	1 (0.6)		
	Strong	3 (1.3)	1 (4.5)	2 (4.5)	0 (0.0)		
**BTLA (%)**	Negative	209 (89.3)	15 (68.2)	33 (75.0)	161 (95.8)	<0.001	Fisher’s exact test
	Weak	20 (8.5)	5 (22.7)	8 (18.2)	7 (4.2)		
	Moderate	2 (0.9)	1 (4.5)	1 (2.3)	0 (0.0)		
	Strong	3 (1.3)	1 (4.5)	2 (4.5)	0 (0.0)		
**TIM3 (%)**	Negative	158 (67.5)	3 (13.6)	19 (43.2)	136 (81.0)	<0.001	Chi-square test
	Weak	41 (17.5)	3 (13.6)	9 (20.5)	29 (17.3)		
	Moderate	17 (7.3)	6 (27.3)	8 (18.2)	3 (1.8)		
	Strong	18 (7.7)	10 (45.5)	8 (18.2)	0 (0.0)		

### Correlation of Immune Checkpoints With Clinicopathological Characteristics

Generally, there was no significant correlation between most ICP expressions and clinical parameters in the three pathologic types (**Supplementary Figure S1**). Regarding 168 PTC patients, only VISTA expression was positively related to age and TNM stage (**Supplementary Figure S1A**). Whereas in the PDTC group (n = 44), the expressions of TIGIT, VISTA, and TIM3 were correlated to older age (**Supplementary Figure S1B**). And higher TNM stage was associated with four ICP expressions including PDL1, TIGIT, VISTA, and TIM3 (**Supplementary Figure S1B**). In addition, male patients with ATC were observed to have a relationship with positive expression of PDL1 and LAG3, and younger patients with ATC tended to exhibit positive status of TIGIT (**Supplementary Figure S1C**).

### Survival Analyses of Immune Checkpoints

In this study, 220 patients were included in survival analyses after 2 ATCs, 3 PDTCs, and 6 PTCs with T4 stage, 2 PTCs with T1 stage, and 1 PTC with T3 stage were removed following the methods described above. Different pathological types showed obviously different OS (p < 0.0001) (**Supplementary Figure S2**). ATC indicated an extremely poor prognosis where most patients died in the first year (**Supplementary Figure S2**). The OS of PDTC lied between ATC and PTC, suggesting an intermediate aggressiveness of tumor. And T4-stage PTCs tended to have poorer prognosis compared with PTCs in T1 stage to T3 stage (**Supplementary Figure S2**).

**Figure 3:**
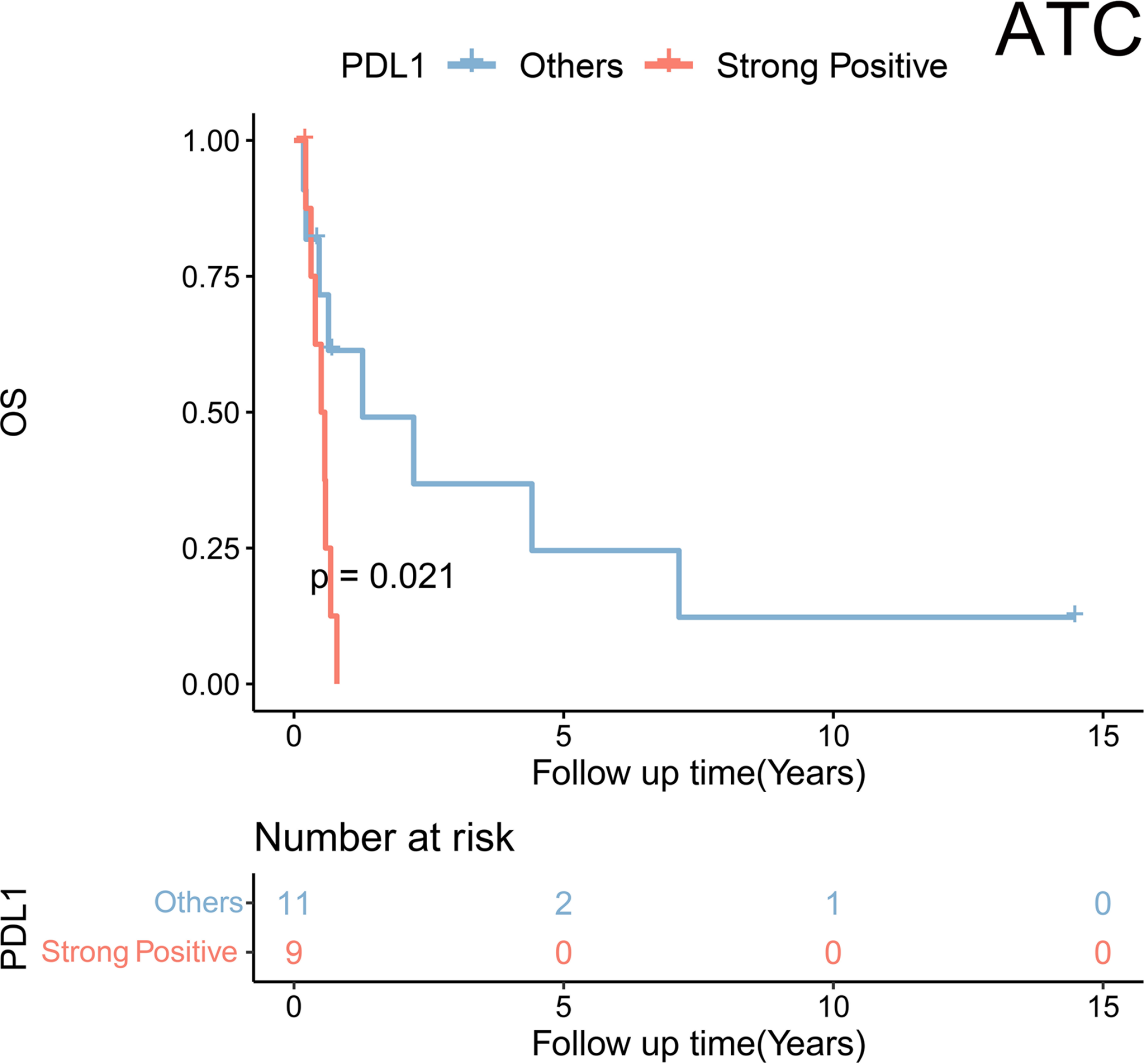
Kaplan–Meier analysis showed a significant difference in overall survival stratified by PDL1 expression status (strong positive vs. non-strong positive) in anaplastic thyroid carcinoma (ATC).

Further analyses were performed in subgroups of TC. First, regarding the ATC, only PDL1 expression was statistically related to a poorer prognosis [strong vs. negative and weak and moderate: hazard ratio (HR) = 3.88, p = 0.031] according to the univariate Cox regression (**Table 3**). KM plot also showed that positive PDL1-expressing ATC cases had much poorer OS than those with negative expression of PDL1 (**Figure 3**).

**Table 3:** Survival analysis of prognostic factors for overall survival in 20 ATCs^*^.

Characteristics	HR	p	95% CI
**Gender Men vs. Women**	2.27	0.137	(0.77–6.68)
**Age (years)**	1.01	0.841	(0.94–1.07)
**T4 vs. T2/T3**	0.43	0.166	(0.13–1.42)
**N1 vs. N0**	1.57	0.493	(0.43–5.7)
**M1 vs. M0**	0.87	0.814	(0.27–2.76)
**PDL1 Strong vs. Negative and Weak and Moderate**	3.88	**0.031**	(1.13–13.24)
**PD1 Positive vs. Negative**	0.46	0.187	(0.15–1.45)
**TIGIT Positive vs. Negative**	1.29	0.635	(0.45–3.66)
**LAG3 Positive vs. Negative**	1.57	0.387	(0.56–4.36)
**VISTA Strong vs. Negative and Weak and Moderate**	1.87	0.351	(0.5–6.98)
**B7H3 Strong vs. Negative and Weak and Moderate**	0.86	0.819	(0.24–3.14)
**CTLA4 Moderate and Strong vs. Negative and Weak**	0.51	0.298	(0.14–1.81)
**BTLA Positive vs. Negative**	0.48	0.207	(0.15–1.5)
**TIM3 Strong vs. Negative and Weak and Moderate**	1.53	0.413	(0.55–4.2)

*Two patients were excluded (one was lost to follow-up and one had followed up in less than 1 month). The bold values indicates that the p values are less than 0.05, which is considered statistically significant.

**Figure 4:**
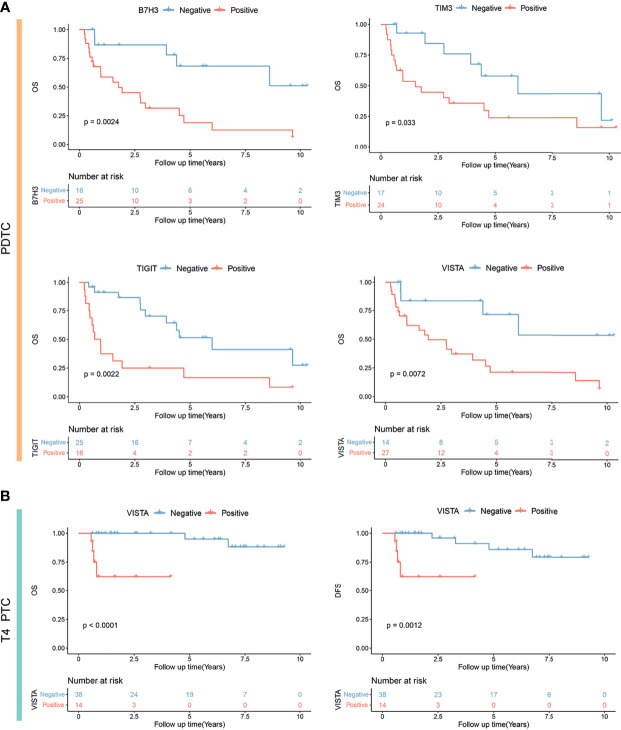
Kaplan–Meier analysis showing significantly different prognoses in poorly differentiated thyroid carcinoma (PDTC) **(A)** and T4-stage papillary thyroid carcinoma (PTC) **(B)** stratified by B7H3, TIM3, TIGIT, and VISTA expression status. OS, overall survival; DFS, disease-free survival.

Second, in the cohort of PDTC, univariate Cox regression was performed to identify the risk factor of survival. It was found that older age (HR = 1.03, p = 0.041), T stage (T3/T4 vs. T1/T2: HR = 4.01, p = 0.024), M stage (M1 vs. M0: HR = 3.62, p = 0.024), TIGIT expression (positive vs. negative: HR = 3.25, p = 0.004), VISTA expression (strong vs. negative and weak and moderate: HR = 3.22, p = 0.007), B7H3 expression (strong vs. negative and weak and moderate: HR = 3.57, p = 0.007), and TIM3 expression (strong vs. negative and weak and moderate: HR = 4.83, p < 0.001) were associated with a poorer prognosis (**Table 4**). Furthermore, multivariate analysis indicated three independent prognostic predictors of PDTC including M stage (M1 vs. M0: HR = 14.45, p < 0.001), B7H3 expression (strong vs. negative and weak and moderate: HR = 12.14, p = 0.0025), and TIM3 expression (strong vs. negative and weak and moderate: HR = 8.25, p < 0.001) (**Table 4**). KM plots visualized that the positive expression groups of B7H3, TIM3, TIGIT, and VISTA had a significantly unfavorable OS in PDTC (**Figure 4A**).

**Table 4:** Survival analysis of prognostic factors for overall survival in 41 PDTCs^*^.

Characteristics	Univariate analysis			Multivariate analysis		
	HR	p	95% CI	HR	p	95% CI
**Gender Men vs. Women**	0.87	0.733	(0.39–1.95)			
**Age (years)**	1.03	**0.041**	(1–1.06)	1.02	0.3222	(0.98–1.06)
**T3/T4 vs. T1/T2**	4.01	**0.024**	(1.2–13.43)	0.46	0.4653	(0.06–3.69)
**N1 vs. N0**	2.8	0.098	(0.83–9.49)			
**M1 vs. M0**	3.62	**0.024**	(1.19–11.04)	14.45	**<0.001**	(3.23–64.7)
**PDL1 Positive vs. Negative**	1.57	0.303	(0.67–3.69)			
**PD1 Positive vs. Negative**	0.92	0.91	(0.21–3.96)			
**TIGIT Positive vs. Negative**	3.25	**0.004**	(1.46–7.21)	1.76	0.266	(0.65–4.79)
**LAG3 Positive vs. Negative**	1.14	0.794	(0.42–3.08)			
**VISTA Strong vs. Others**	3.22	**0.007**	(1.38–7.53)	0.68	0.5428	(0.2–2.33)
**B7H3 Strong vs. Others**	3.57	**0.007**	(1.41–8.99)	12.14	**0.0025**	(2.41–61.14)
**CTLA4 Positive vs. Negative**	2.04	0.08	(0.92–4.54)			
**BTLA Positive vs. Negative**	1.29	0.584	(0.51–3.25)			
**TIM3 Strong vs. Others**	4.83	**<0.001**	(2.03–11.51)	8.25	**<0.001**	(2.4–28.31)

*Three patients were excluded (two were lost to follow-up and one had followed up in less than 1 month). The bold values indicates that the p values are less than 0.05, which is considered statistically significant.

Third, univariate Cox analysis was conducted in the subgroup of 52 locally advanced PTCs (pT4 stage). It was observed that older age (HR = 1.15, p = 0.0150), M stage (M1 vs. M0: HR = 36.5, p = 0.001), and VISTA expression (positive vs. negative: HR = 13.79, p = 0.025) were risk factors of OS (**Table 5**). Moreover, M stage (M1 vs. M0: HR = 29.22, p = 0.0065) and VISTA expression (positive versus negative: HR = 42.23, p = 0.021) were independent predictors of OS in PTC patients with locally advanced disease (**Table 5**). Similarly, older age (HR = 1.15, p = 0.0150), M stage (M1 vs. M0: HR = 36.5, p = 0.001), VISTA expression (positive vs. negative: HR = 13.79, p = 0.025), and B7H3 expression (moderate and strong vs. weak and negative: HR = 6.43, p = 0.027) were risk factors of DFS (**Table 5**), and independent predictors of DFS were still M stage (M1 vs. M0: HR = 32.85, p = 0.007) and VISTA expression (positive vs. negative: HR = 12.94, p = 0.022). TIM3 positivity was not associated with a longer OS in locally advanced PTC. Subsequently, significant different prognoses were illustrated by dividing the locally advanced PTC set into negative and positive expression groups of VISTA and B7H3 (**Figure 4B** and **Supplementary Figure S3**).

**Table 5:** Survival analysis of prognostic factors for overall survival and disease-free survival in locally advanced 52 PTCs (pT4)^*^.

Overall survival						
Characteristics	Univariate analysis			Multivariate analysis		
	HR	95%CI	p	HR	95%CI	p
**Gender Male vs. Female**	0.41	(0.08-2.28)	0.3100			
**Age (years)**	1.15	(1.03-1.28)	**0.0150**	1.13	(0.96-1.33)	0.1384
**Tumor Size (cm)**	0.85	(0.48-1.48)	0.5560			
**N1b vs. N0/N1a**	2.3	(0.27-19.7)	0.4480			
**M1 vs. M0**	36.5	(4.24-313.91)	**0.0010**	29.22	(2.57-331.79)	**0.0065**
**TIGIT Positive vs. Negative**	8.81	(0.91-85.62)	0.0610			
**LAG3 Positive vs. Negative**	1.27	(0.15-10.88)	0.8290			
**VISTA Moderate & Strong** **vs. Weak & Negative**	13.79	(1.4-135.91)	**0.0250**	42.23	(1.76-1013.87)	**0.0210**
**B7H3 Moderate & Strong** **vs. Weak & Negative**	3.53	(0.63-19.93)	0.1530			
**CTLA4 Positive vs. Negative**	1.39	(0.16-11.91)	0.7650			
**TIM3 Positive vs. Negative**	1.96	(0.36-10.7)	0.4380			
**Disease Free Survival**						
**Characteristics**	**Univariate analysis**			**Multivariate analysis**		
	**HR**	**95%CI**	**p**	**HR**	**95%CI**	**p**
**Gender Male vs. Female**	0.98	(0.24-3.96)	0.9760			
**Age (years)**	1.12	(1.02-1.22)	**0.0140**	1.04	(0.94-1.15)	0.4567
**Tumor Size (cm)**	0.88	(0.56-1.4)	0.6020			
**N1b vs. N0/N1a**	1.34	(0.27-6.65)	0.7230			
**M1 vs. M0**	24.78	(4.98-123.44)	**<0.001**	32.85	(2.6-415.45)	**0.0070**
**TIGIT Positive vs. Negative**	6.57	(0.73-59.2)	0.0930			
**LAG3 Positive vs. Negative**	0.92	(0.11-7.47)	0.9340			
**VISTA Positive vs. Negative**	5.64	(1.1-28.99)	**0.0380**	12.94	(1.45-115.73)	**0.0220**
**B7H3 Moderate & Strong** **vs. Weak & Negative**	6.43	(1.24-33.39)	**0.0270**	0.55	(0.06-5.08)	0.6004
**CTLA4 Positive vs. Negative**	0.93	(0.11-7.59)	0.9480			
**TIM3 Positive vs. Negative**	1.24	(0.25-6.14)	0.7960			

*Six patients were lost to follow-up. The bold values indicates that the p values are less than 0.05, which is considered statistically significant.

### Co-Expression of Immune Checkpoints in Thyroid Cancer

**Figure 5:**
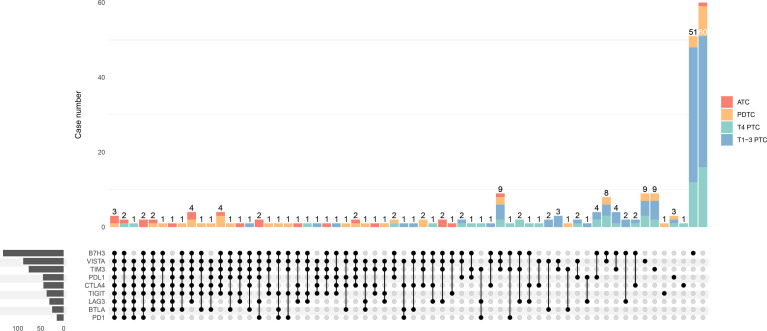
Upset plot visualizing the co-expression profiles of immune checkpoints in thyroid carcinoma, including anaplastic thyroid carcinoma (ATC), poorly differentiated thyroid carcinoma (PDTC), T4-stage papillary thyroid carcinoma (T4 PTC), and T1-3-stage PTC (T1-3 PTC). Each row corresponds to an immune checkpoint (ICP) with positive expression of which the bar charts on the left show the size. Each column corresponds to a co-expression status: the filled-in cells show which ICP is co-incidence. The bar charts on the top indicates the case number of the co-expression status where ATC, PDTC, T4 PTC, and T1-3 PTC are filled with red, orange, green, and blue, respectively. The left side reveals that most cases with a great number of co-expressed ICPs are ATC (red) and PDTC (orange), whereas cases with much less co-positivity of ICPs are T4 PTC (green) and T1-3 PTC (blue) on the right side.

The particular co-expression profile of the entire cohort was visualized (**Figure 5**). Briefly, each row corresponded to a positive expression of ICPs, and bar charts on the left showed the size of the set. Each column corresponded to a co-expression status: the filled-in cells show which ICP was co-incidence. The bar charts on the top indicated the size of the co-expression status where different subtypes of TC were filled with different colors. It was demonstrated that most PTC tumors exhibited no or single ICP expression, whereas most ATC tumors had co-expression of a considerable number of ICPs. Meanwhile, PDTC demonstrated a heterogeneity of ICP co-expression (**Figure 5**). According to the results from the previous univariate and multivariate analyses, independently predictive ICPs were conducted into further co-expression analysis in subsets of PDTC and locally advanced PTC.

**Figure 6:**
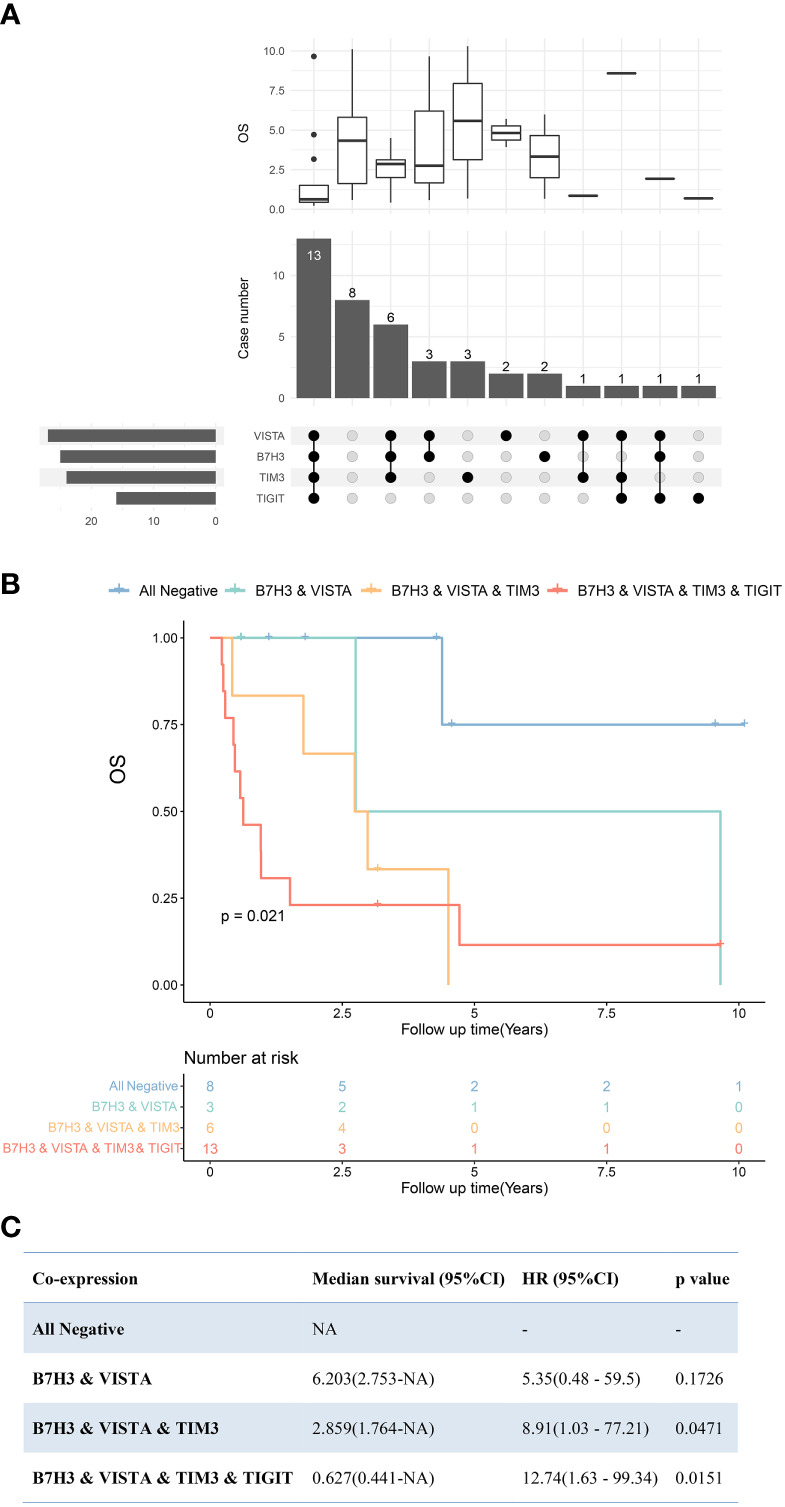
Upset plot visualizing the co-expressive profiles of VISTA, B7H3, TIM3, and TIGIT in poorly differentiated thyroid carcinoma (PDTC). **(A)** Thirteen PDTCs expressed VISTA combined with B7H3, TIM3, and TIGIT; 8 PDTCs expressed none of VISTA, B7H3, TIM3, and TIGIT; 3 PDTCs expressed VISTA together with B7H3; and 6 expressed VISTA together with B7H3 and TIM3. The boxplot above depicting the median survival of the immune molecule combinations depicted in the upset plot below. **(B)** Kaplan–Meier plot of PDTC patients with VISTA-, B7H3-, TIM3-, and TIGIT-, B7H3-, VISTA- and TIM3-, B7H3- and VISTA-expressing tumors, as well as those with no expression of these four molecules (p = 0.021). **(C)** Cox regression revealed median survivals of co-expression PDTC subsets and the hazard ratio (HR) with 95% confidence interval (95% CI) compared with all negative PDTC subsets. NA, not applicable.

In terms of PDTC, VISTA (64%) was the most frequently expressed immune molecule among four ICPs that were observed as risk predictors of OS previously, followed by B7H3 (59%), TIM3 (57%), and TIGIT (39%) (**Figures 2**, **6A** and **Table 2**). Thirteen PDTC tumors expressed VISTA combined with B7H3, TIM3, and TIGIT; three tumors expressed VISTA together with B7H3; and six tumors expressed VISTA together with B7H3 and TIM3 (**Figures 6A, B****)**. Meanwhile, eight PDTC tumors had all negativity of VISTA, B7H3, TIM3, and TIGIT (**Figure 6A**). The survival of the four largest groups differed significantly (all negative, VISTA and B7H3, VISTA together with B7H3 and TIM3, and VISTA together with B7H3, TIM3, and TIM3), with a poorer outcome found as the number of co-positivity rose (p = 0.021, **Figure 6B**). The median survival of PDTC subgroups with B7H3/VISTA, B7H3/VISTA/TIM3, and TIGIT/B7H3/VISTA/TIM3 co-expression was 6.203 years, 2.859 years, and 0.627 years, respectively. Median survival time was not observed in the non-expressed TIGIT/B7H3/VISTA/TIM3 subset. Compared with the control PDTC subgroup (all negative), HR of B7H3/VISTA, B7H3/VISTA/TIM3, and TIGIT/B7H3/VISTA/TIM3 co-expression was 5.35 (95% CI: 0.47–59.5, p = 0.1726), 8.91 (95% CI: 1.03–77.21, p = 0.0471), and 12.74 (95% CI: 1.63–99.34, p = 0.0151) (**Figure 6C**).

**Figure 7:**
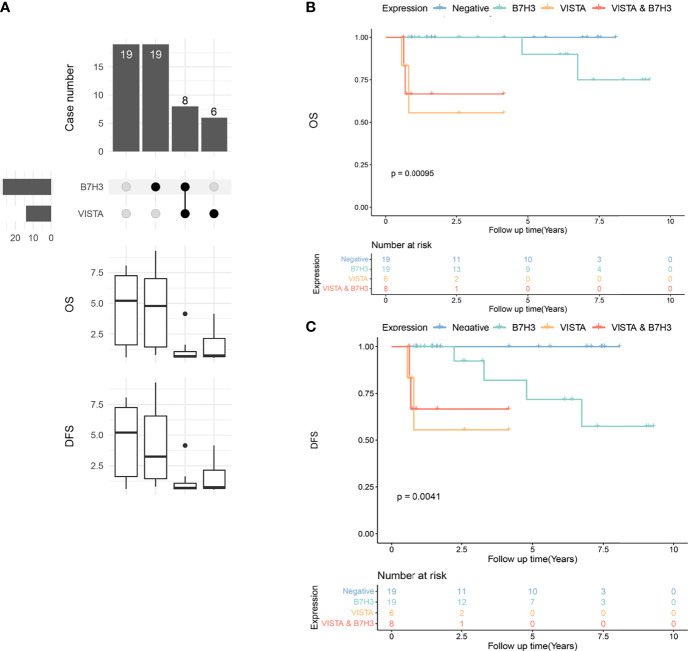
Upset plot visualizing the co-expressive profiles of VISTA and B7H3 in advanced papillary thyroid carcinoma (PTC). **(A)** Nineteen PTCs have no positivity of B7H3 and VISTA, 19 PTCs expressing B7H3 alone, 8 PTCs expressing B7H3 and VISTA, and 6 PTCs expressing VISTA alone. The boxplot below depicting the median overall survival (OS) and disease-free survival (DFS) of the immune molecule combinations depicted in the upset plot above. **(B)** Kaplan–Meier plot of OS in PTC patients with VISTA-, B7H3-, B7H3- and VISTA-expressing tumors, as well as those with no expression of these two molecules (p = 0.00095). **(C)** Kaplan–Meier plot of DFS in PTC patients with VISTA-, B7H3-, B7H3- and VISTA-expressing tumors, as well as those with no expression of these two molecules (p = 0.0041).

B7H3 and VISTA were two ICPs with the highest positive-expressing ratios in PTC and locally advanced PTC (**Figures 2**, **7A**). There were 19 tumors expressing B7H3 alone, six expressing VISTA alone, and eight tumors expressing B7H3 combined with VISTA (**Figure 7A**). In the meantime, 19 tumors had no expression of both VISTA and B7H3 (**Figure 7A**). The OS and DFS showed significant differences among the four subgroups (negative, B7H3, VISTA, VISTA and B7H3), indicating distinctive abilities of these two ICPs, especially positive expression of VISTA (**Figures 7B, C****)**.

## Discussion

To the best of our knowledge, this was the first study to assess the expression levels of nine ICPs in a large cohort of advanced TC patients, with a focus on ATCs, PDTCs, and locally advanced PTCs. Prognostic analyses of ICIs were performed in order to support personalized precision medical plans for advanced TC patients of various pathological types. In recent decades, studies have shown that TIICs have dual roles, either promoting or inhibiting tumor development through complex factors (30). Specific immunotherapy can be achieved by regulating inhibitory or stimulatory checkpoints with relative blockades. However, a great number of patients develop resistance or nonresponse. No curative outcome was observed in most cases of advanced TC during ICP monotherapies. For instance, one study applying anti-PD1 combined with kinase inhibitors for ATC patients reported that no patient achieved a complete response, and five out of 12 achieved a partial response (11). Another study reported a 19% overall response rate to anti-PD1 monotherapy in progressive ATC (12). Accumulating evidence indicates an urgent need to improve the response to immunotherapies. In addition to traditional ICPs, many “next-generation” molecules targeting other well-established immune-regulatory proteins are being tested, and more data are needed to support and guide clinical treatment.

The expression of ICP in the tumor microenvironment (TME) reveals the level of internal immunomodulation and also provides prognostic information. The incidence and intensity of ICP expression were significantly higher in ATC than in WDTC (31). For example, the PDL1-positive rate in ATC, PDTC, and PTC was 64%, 32%, and 10%, respectively, which was consistent with previous studies (32–35).

PDL1 was observed to be an ATC prognostic biomarker (**Figure 3**), which provided strong evidence of clinical application of anti-PDL1 molecules in ATC once again. Except for PDL1, there were no statistically significant differences in OS between ATC and the other eight ICPs. This could be due to the extremely short survival of ATC patients and the small sample size, making it difficult to distinguish the prognoses. Interestingly, a strong positive correlation was discovered between men and the expression of PDL1 and LAG3 in ATC patients (**Supplementary Figure S1C**). And a meta-analysis of 11,351 individuals with advanced or metastatic malignancies discovered that male patients treated with ICIs had significantly longer OS than female patients when compared to their own control group (36). We presumed that ICIs, particularly PDL1 and LAG3, might be more effective for male ATC patients than female ATC patients. Besides, the relatively high rate of positive expression of the other eight ICPs, with the exception of PDL1, still suggested a potential clinical application in ATC, and more clinical trials are needed. In the future, ATC patients may benefit from combination immunotherapy centered on PD1/PDL1.

ICIs should improve survival if low survival is linked to the corresponding checkpoint molecules. In our study, the expression profile of ICPs in PDTC was found to be highly heterogeneous. Patients who co-expressed VISTA, TIM3, B7H3, and TIGIT had a significantly worse prognosis than those who did not express any of these four ICPs (**Figure 7B**). The findings revealed that PDTC patients with more than two, or even four, positively expressed VISTA, TIM3, B7H3, and TIGIT had significantly lower survival. Thus, a combination of VISTA, TIM3, B7H3, or TIGIT blockers might help improve the clinical outcome of PDTC patients with high levels of ICP co-expression, where these costimulatory signals may have a significant synergistic effect. More studies are necessary as a result of this discovery.

PTC patients, on the other hand, had a relatively “cold” property. Recently, a low response to anti-PD1 therapy was observed in patients with advanced WDTC, with 2 of 22 cases confirmed as partial response (13). In this study, B7H3 and VISTA were discovered to be the two most frequently expressed ICPs in PTC, with 52% and 23% positivity ratios, respectively (**Figure 2A**). According to the multivariate analysis, VISTA expression was an independent risk factor in our subset of locally advanced PTC, and patients with co-positivity of VISTA and B7H3 also had an unfavorable prognosis, despite the fact that B7H3 was not an independent factor of OS or DFS. Our findings suggest that VISTA inhibitors might benefit advanced PTC patients and that the inhibition of VISTA in combination with B7H3 might be a novel meaningful supplement to PD1/PDL1 signaling monotherapy in these patients.

VISTA is a new ICP found in a variety of cancers (37–39). In this study, most ATC and PDTC cases expressed VISTA, and a high proportion of the co-expression of VISTA with other ICPs was demonstrated in ATC. For instance, positive expression of VISTA together with PDL1 and/or PD1 was observed in more than half of ATC cases (VISTA and PDL1: n = 14/22; VISTA and PD1: n = 7/22; VISTA and PD1/PDL1: n = 15/22) in our study. The association between VISTA and PD1/PDL1 signaling had been found, implying a probable shared mechanism (40). Another study mechanically showed a nonredundant role of VISTA, which was distinct from the PD1/PDL1 pathway in controlling T-cell activation (41). B7H3 is involved in the inhibition of T cells, is overexpressed in a wide spectrum of tumor tissues, and is linked to disease states and prognosis (42, 43). In this study, more than half of PTC tumors expressed B7H3 positively, of which the rate was much higher than other ICP expressions in PTCs. We speculated that the response rate of anti-B7H3 could be high in advanced TC based on its high expression. Furthermore, B7H3 and PDL1 play nonredundant and somewhat complementary roles in tumor immune evasion (44). Another new ICP, TIM3, contributes to the dampening of protective immunity, specifically limiting the responses of Th1 and Tc1 T cell (45, 46). Interestingly, TIM3 was upregulated most notably among several ICPs in T cells from patients who developed resistance to anti-PD1 treatment (47). This provided evidence that the failure of immunotherapeutics was associated with upregulation of alternative immunosuppressive ICPs.

Our study was not without limitations. First, given the rarity of ATC and PDTC, our large single-center study is still limited by sample size, and further studies from multiple centers are required to support our findings. Second, several clinically significant gene mutations in thyroid cancer (such as *BRAF*, *RAS*, *TP53*, and *TERT*) were not accessed. Their relationships with ICPs should be evaluated in our future study. Last, the intrinsic mechanisms of ICPs in advanced TC are still under investigation, and more research is required.

## Conclusion

We are the first to show the landscape of a large number of ICPs in a large cohort of advanced TCs. PDL1 expression was associated with poorer ATC survival, and ATC had a high level of ICP co-expression, supporting PDL1’s prognostic and therapeutic value and implying a prospective PD1/PDL1-centered combination immunotherapy in ATC. B7H3, TIM3, VISTA, and TIGIT expressions were prognostic biomarkers of PDTC. Furthermore, the number of co-positivity of these four ICPs was linked to the OS in PDTC patients, implying that combining VISTA, TIM3, B7H3, or TIGIT blockers might help improve survival. Finally, VISTA may be a potential immune predictor of prognosis in locally advanced PTC, and we proposed that VISTA monotherapy or anti-VISTA in combination with an anti-B7H3 agent could show promising results.

## Data Availability Statement

The original contributions presented in the study are included in the article and supplementary material. Further inquiries can be directed to the corresponding authors.

## Ethics Statement

The studies involving human participants were reviewed and approved by the ethics committee of Fudan University Shanghai Cancer Center. The patients/participants provided their written informed consent to participate in this study.

## Author Contributions

YL: Study design, data acquisition, data analysis, article writing;Y-CY: Data acquisition; C-KS: Data acquisition; BM: Data acquisition; W-BX: Data analysis; Q-FW: Data acquisition; YZ: Data acquisition; TL: Data acquisition; W-JW: Study design and methodology, article writing; YW: Study design and review. All authors contributed to the article and approved the submitted version.

## Funding

Yu Wang: National Natural Science Foundation of China (82072951), Science and Technology Commission of Shanghai Municipality (19411966600), Shanghai Anticancer Association (SACA-AX106), Shanghai Shen-Kang Hospital Development Center (SHDC2020CR6003). Wen-Jun Wei: Natural Science Foundation of Shanghai (22ZR1412800).

## Conflict of Interest

The authors declare that the research was conducted in the absence of any commercial or financial relationships that could be construed as a potential conflict of interest.

## Publisher’s Note

All claims expressed in this article are solely those of the authors and do not necessarily represent those of their affiliated organizations, or those of the publisher, the editors and the reviewers. Any product that may be evaluated in this article, or claim that may be made by its manufacturer, is not guaranteed or endorsed by the publisher.
