# Supplementary material for: Immune Checkpoint Protein Expression Defines the Prognosis of Advanced Thyroid Carcinoma
Source: Front Endocrinol (Lausanne). 2022 Apr 27;13:859013. doi: 10.3389/fendo.2022.859013 (PMC9094437; doi:10.3389/fendo.2022.859013)
Supplement: Supplementary file 1 [file DataSheet_1.docx]

Supplementary Material

# Supplementary Figures

#
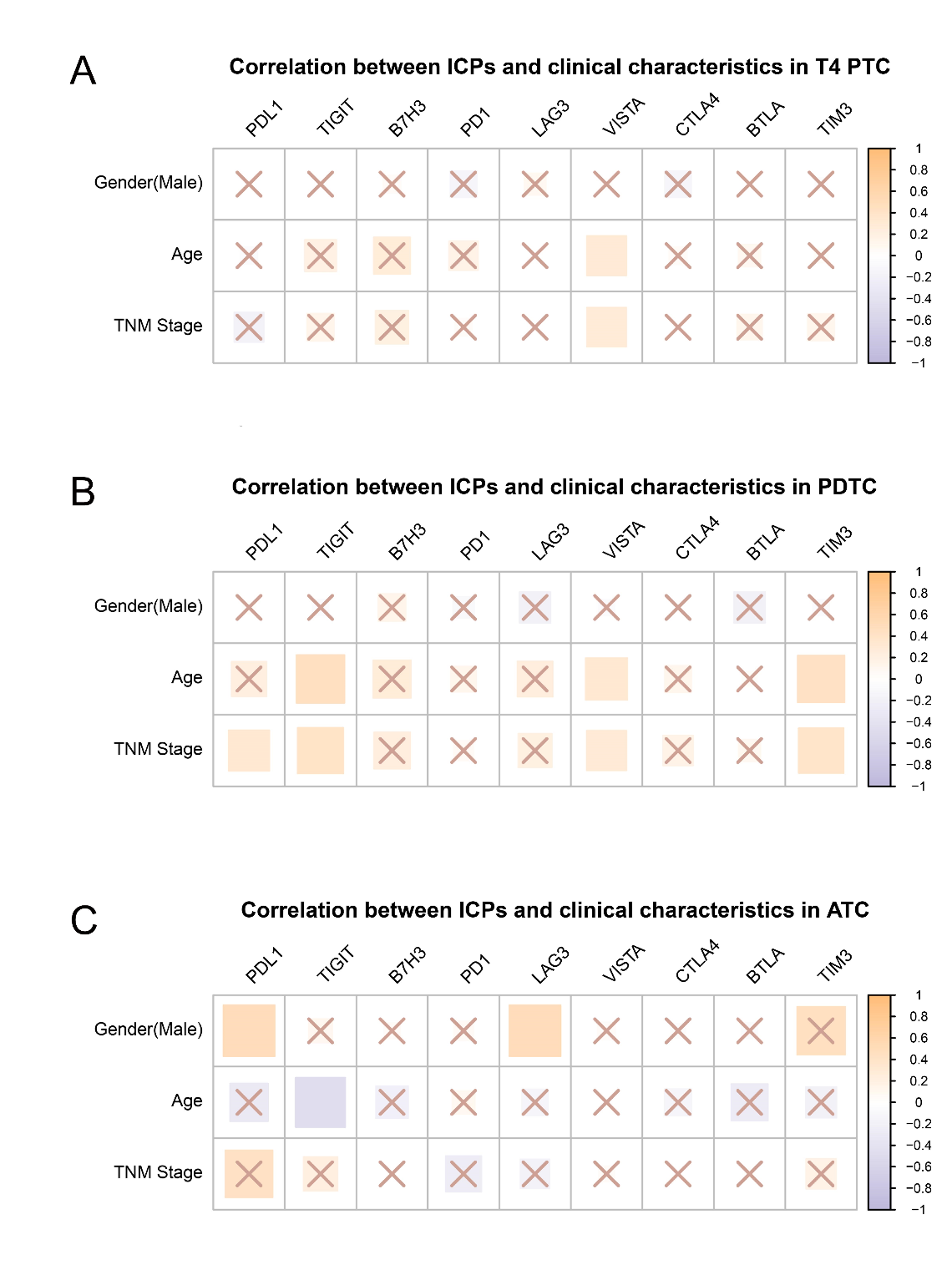


**Supplementary Figure 1.**

Spearman correlation between clinical factors and immune checkpoints expression in cohort of T4 stage papillary thyroid carcinoma (PTC) (A), poorly differentiated thyroid carcinoma (PDTC) (B), and anaplastic thyroid carcinoma (ATC) (C). TNM stage of PTC and PDTC included I, II, III, IV stage, whereas the TNM stage of ATC included IVA, IVB, IVC stage. “X” represents p value ≥ 0.05.


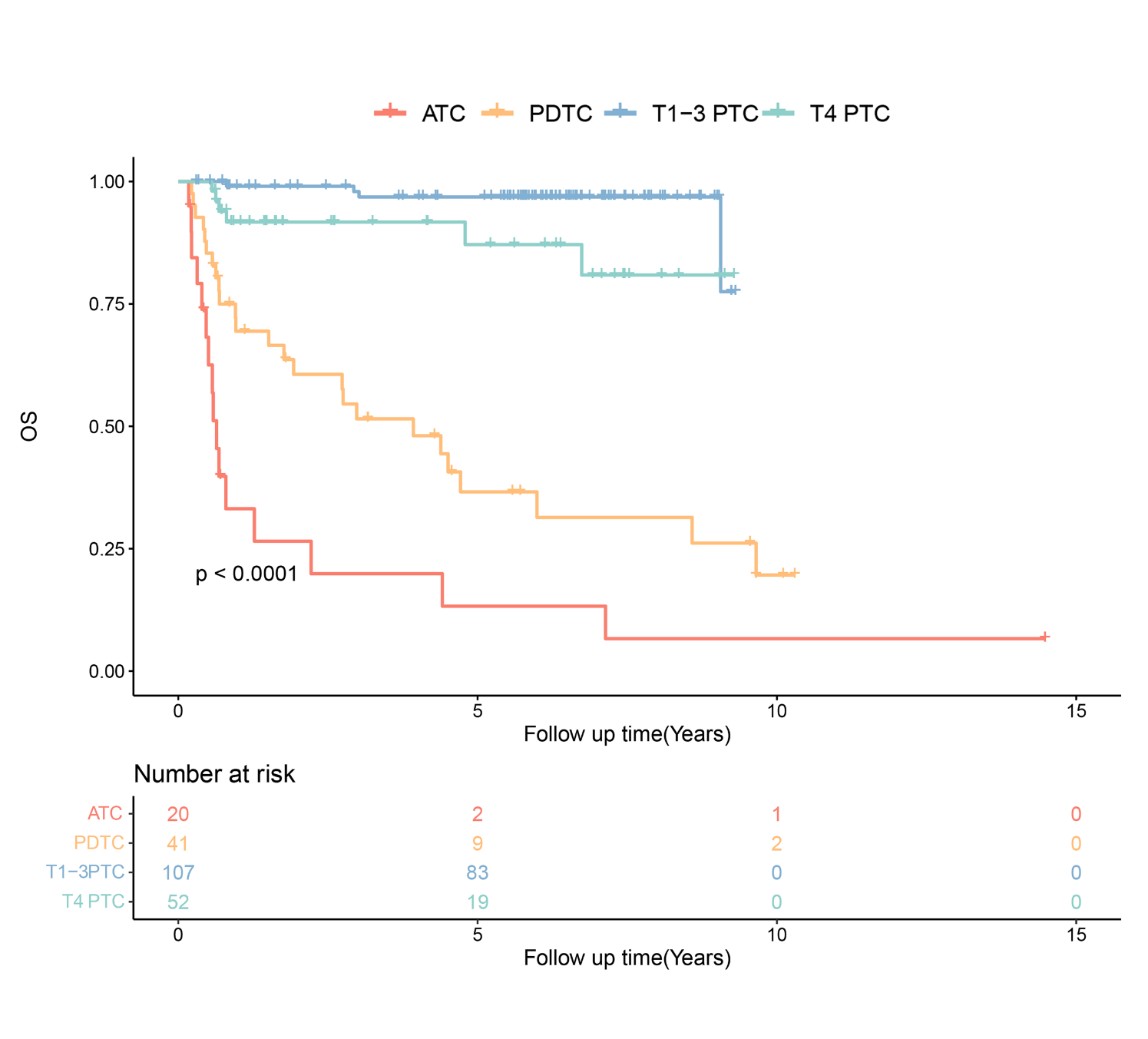


**Supplementary Figure 2.** Kaplan-Meier analysis showing differences in overall survival stratified by pathological type.


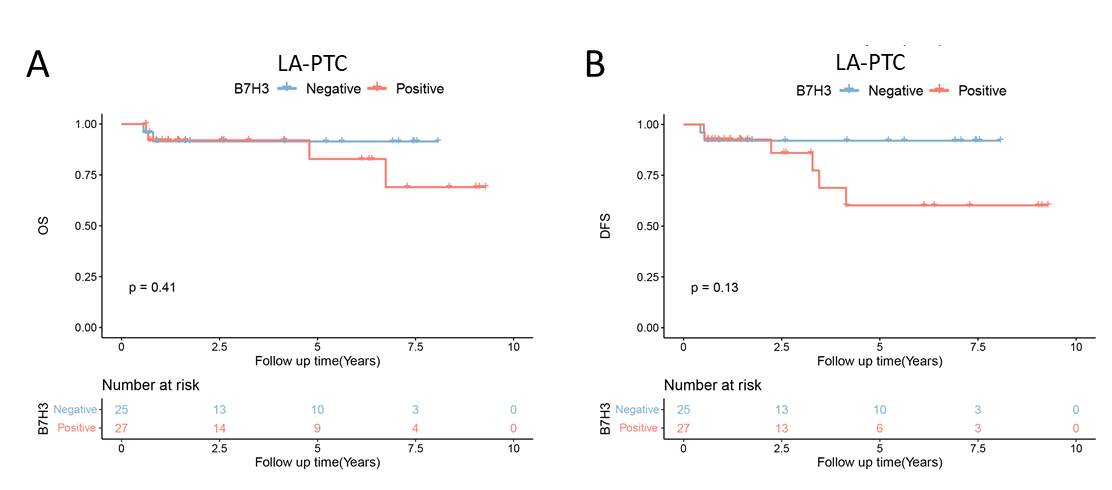


**Supplementary Figure 3.** Kaplan-Meier analysis showing a significant difference in overall survival (OS) and disease-free survival (DFS) stratified by B7H3 expression status in locally advanced PTC (LA-PTC).

# Supplementary Table

**Supplementary Table S1. Antibodies applied for immunohistochemistry**

| Antibodies | Source | Catalog No. | Dilution |
| --- | --- | --- | --- |
| Anti-PD1 antibody | Abcam, Cambridge, UK | ab52587 | 1:200 |
| Anti-CTLA4 antibody | Abcam, Cambridge, UK | ab237712 | 1:500 |
| Anti-TIM3 antibody | Abcam, Cambridge, UK | ab241332 | 1:500 |
| Anti-LAG3 antibody | Abcam, Cambridge, UK | ab209236 | 1:200 |
| Anti-TIGIT antibody | Abcam, Cambridge, UK | ab243903 | 1:200 |
| Anti-BTLA antibody | Abcam, Cambridge, UK | ab230976 | 1:100 |
| Anti-VISTA antibody | Abcam, Cambridge, UK | ab230950 | 1:100 |
| Anti-PDL1 antibody | Cell Signaling Technology, MA, USA | E1L3N | 1:200 |
| Anti-B7H3 antibody | Cell Signaling Technology, MA, USA­ | D9M2L | 1:200 |
